# Laser-PIXE using laser-accelerated proton beams

**DOI:** 10.1038/s41598-019-42997-y

**Published:** 2019-05-02

**Authors:** M. Barberio, P. Antici

**Affiliations:** INRS-EMT, 1650 boul, Lionel-Boulet, Varennes, QC J3X 1S2 Canada

**Keywords:** High-field lasers, Characterization and analytical techniques, Plasma-based accelerators

## Abstract

Laser-driven proton acceleration is a field of growing interest, in particular for its numerous applications, including in the field of materials science. A benefit of these laser-based particle sources is their potential for a relative compactness in addition to some characteristics at the source that differ from those of conventional, radio-frequency based proton sources. These features include, *e.g*., a higher brilliance, a shorter duration, and a larger energy spread. Recently, the use of laser-accelerated protons has been proposed in the field of Cultural Heritage, as alternative source for the Particle Induced X-ray Emission diagnostic (“laser-PIXE”), a particular ion beam analysis (IBA) technique that allows to precisely analyse the chemical composition of the material bulk. In this paper we study the feasibility of the laser-PIXE using laser-accelerated proton beams. We focus on materials specifically of interest for the Cultural Heritage domain. Using Geant4 simulations, we show that the laser-PIXE allows analysing a larger volume than conventional PIXE, profiting from the large energy spread of laser-accelerated protons. Furthermore, for specific materials, the large energy spread allows investigating multilayer materials, providing an advantage compared to conventional PIXE technologies.

## Introduction

In the last decade, laser-driven particle acceleration, obtained by interaction of a high-power laser with a target, has become a rapidly expanding field due to the unique characteristics of these beams. Many researchers working in different infrastructures are currently investigating potential applications where these laser-generated beams can outperform conventionally generated particles^1^ and become usable laser-based particle beamlines^2–9^. Recently, strong attention has been devoted to applications in materials science, with some pioneering works published recently^10–15^. This includes investigations associated with Cultural Heritage^16^, since the field is currently hampered by the lack of techniques and diagnostics suitable for the conservation and the preservation of artworks or monuments^17,18^. Consequently, there is a strong and urgent need for the discovery and development of new techniques and methodologies^19^ that should either preserve or restore Cultural Heritage. These techniques can be twofold: (1) techniques that are cheap(er) and able to be widely applicable *e.g.*, by museums or restoration centres; and (2) techniques that are able to solve very specific issues that cannot be solved with conventional diagnostic techniques, and where any means (including financial) would be acceptable if they allow overcoming important bottlenecks^20^, especially considering the very high value of some artifacts^21^.

Very recently, the use of laser-generated protons has been introduced for generating Particle Induced X- and Gamma-ray Emission spectroscopy (PIXE/PIGE) from materials (laser-PIXE)^11^. PIXE (in the following we will refer only to PIXE, but all information is applicable also for PIGE) is a widespread technique in materials science, in particular ion-beam analysis. The technique allows obtaining a complete chemical analysis of the material’s bulk, with a micrometric spatial resolution, thus distinguishing the corrosive patina from the main components of the artifacts. Compared to its strongest competitor, the X-Ray Fluorescence (XRF) technique, PIXE has, *e.g*., the advantage of allowing analysing very small samples and has a higher sensitivity (tens of parts per millions). Publications comparing both techniques indicate that PIXE and XRF allow having complementary information, and are not easily mutually replaceable^22,23^, with the significant difference that PIXE facilities, requiring conventional accelerators, are much more expensive than XRF tools and therefore less accessible. Nonetheless, the need of PIXE in the Cultural Heritage explains that many important museums such as The Louvre or Uffizi have dedicated PIXE facilities located very nearby^24^.

In a recent proof-of-principle experiment performing laser-driven PIXE on a silver sample^11^ we demonstrated that laser-accelerated protons allow obtaining a complete chemical analysis of the material’s bulk, with the potential of being quicker and analysing larger surfaces than conventional PIXE facilities. In this experiment we were limited by the sensitivity of the X-ray spectrometer, sensitive only to photons with energy higher than 20 keV, *i.e*., close to the Kα of silver. However, in order to validate our technique, it is crucial to demonstrate, first theoretically and then experimentally, that the laser-driven PIXE is applicable to a large range of materials. Differently than in conventional PIXE, where the proton beam is almost monochromatic and typically ranging from 1–5 MeV, in the laser-driven PIXE the proton source can be very polychromatic. Protons can reach higher energies (laser-generated protons obtained on commercially available lasers range up to tens of MeV) and the proton beam is diverging. As such, the interaction scenario between this kind of source with an artefact is completely different from what occurs on a conventional PIXE accelerator: protons with different energies can produce photons at different depths in the material bulk. However, in order to be detected, these photons need to travel dissimilar distances to escape the material’s bulk. Therefore, whenever signal produced by laser-generated PIXE is detected on a X-ray detector, it is not straightforward to know in which material layer the X-rays have been produced, and what proton energies are required to produce photons that are detectable by a detector positioned at a certain distance from the sample. Similarly, it is questionable up to what extend higher-energy protons (>5 MeV), such as those easily produced by laser-acceleration, can be useful in the laser-PIXE analysis of artifacts. It is important to assess if laser-accelerated proton bunches, by virtue of its broad spectra, can bring depth ‘access’ to the PIXE in its integrated detection.

In this paper, we use Geant4 simulations for investigating the feasibility of laser-driven PIXE on a very large range of materials, in particular for the main chemical elements of importance for the Cultural Heritage context. We demonstrate that in most materials it is possible to obtain a good X-rays detection with, consequently, a very sensitive analysis of materials, and this for a wide range of material depths.

## Simulation Setup

**Figure 1:**
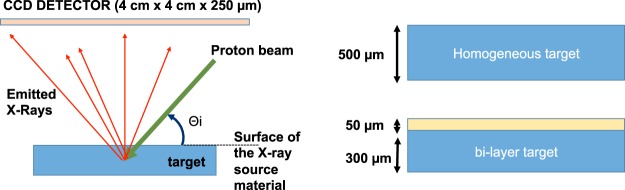
(**a**) Experimental setup of the Geant4 simulations; (**b**) Sketch of the material configuration used in the simulations.

The simulations were performed using the Geant4 software^25^, a toolkit for the simulation of the passage of particles through matter, and reference in the field of particle acceleration. Geant4 provides functionality to simulate PIXE by means of the G4hImpactIonisation package, which simulates the impact of protons with a material and the consequent generation of X-rays (K-L-M line and bremsstrahlung emission). The simulation model is described in detail in refs^26–30^. In this work we used the ionization cross sections distributed with Geant4 as G4PII data set for an energy range from 1 keV to 200 MeV. The set-up considered in the Geant4 toolkit consisted of a material block that was irradiated with protons, where in this case we have modelled as proton source laser-accelerated protons. In the proton-material interaction process, impact ionization of material atoms generates vacancies in atomic shells. These are then filled by atomic de-excitation, resulting in the emission of fluorescence photons and Auger electrons - the PIXE process. The fluorescence photons were detected by a Si CCD (in the simulations the detector had lateral dimensions of 4 × 4 cm with a thickness of 250 µm), positioned at a distance of 4 cm from the surface of the material block. The CCD detected all photons emitted from the front side of the target within a solid angle in the order of 0.81 steradian. The output of the simulations provided information about the energy, in MeV, of every photon that reached the detector (tracker). Figure 1a shows a sketch of the experimental setup implemented in the simulations. All the simulations were performed in vacuum conditions, consistent with typical experimental setups in laser-driven proton acceleration. However, results obtained for an experimental setup in air would be simiar, with the only difference of producing shorter travelling distances for the particles and photons due to different stopping power.

Modelling of the proton beam source was computed with the package G4ParticleGun, used in order to simulate a beam with cylindrical symmetry with a number of particles variable between 10^4^ and 10^13^. The proton beam had a diameter of 1 µm, which is consistent with typical PIXE applications. In most analysis processes the size of the extracted core sample to be analysed is in the micrometer range, since its extraction should be as unobtrusive as possible. We chose a cylindrical symmetry, since this simplified the computational burden and we had seen that considering a diverging proton beam, as obtained during laser-plasma acceleration, only contributed to an extremely negligible modification of the results: in addition, any divergent proton beam profile can easily by reproduced by enlarging the cylindrical proton beam profile. It may be added that laser-accelerated proton beams, given their divergent feature, allow irradiating surfaces of up to cm^2^ at distances of a few cm. However, using pinholes or beam tailoring devices, the surface can easily be adjusted from a few µm^2^ (e.g using a µm pinhole) up to these cm^2^ surface sizes. When considering the total analysed volume in the following simulations, one needs to take into account that the depth considered in the simulations accounts for these surface areas.

The simulations were performed using a variable number of protons, with fixed particle energy ranging between 0.5 MeV and 10 MeV. In Geant4 all particles are simulated as mono-energetic bunches, without any energy spread. The suggested energy range is consistent with typical proton energies that can routinely be obtained using commercial table-top lasers^11^. To cover the entire energy range, the proton spectrum was discretized choosing different energy step-sizes, depending on the material onto which the protons were impinging (“target material/samples”). The step-sizes in the spectrum were chosen between 0.2–0.5 MeV, which corresponds to penetration-depth steps of about 5 µm (this information was obtained using SRIM simulations^31^). Penetration-depth steps of about 5 µm are smaller than what is typically investigated on artifacts, where the steps can be in the order of tens of microns. In Geant4, the energy thresholds for the production of fluorescence photons and Auger electrons by PIXE were set to a value of 250 eV, *i.e*., protons below this value were not producing X-rays. We used two different categories of target samples (see Fig. 1b): a homogenous box of material and a multilayer of different materials. For the homogeneous materials, we used SiO_2_ (as main constituent in ceramic artifacts), silver and bronze. For the multilayer samples we used the combination of a layer of silver positioned on top of a bronze substrate (silver//bronze) and a multilayer of lead oxide//SiO_2_. We chose these materials for their relevance in the field of Cultural Heritage: the main materials studied by classical PIXE are, typically, pottery (SiO_2_ based ceramic materials), noble metals (coins or jewellery in silver or gold), bronzes (typically consisting of a copper 90% and tin 10% alloy), and pictorial layers (*e.g.*, lead white pigments (PbO) on a ceramic artifact or canvas).

The study of multilayer materials is important in many real case studies, for example the analysis of counterfeit coins/jewellery, or the analysis of pigments situated on ceramics and pottery, which often allows retrieving information about the provenance and age. For simplifying this case we simulated a layer of 50 µm of silver on a bulk of 300 µm of bronze, and a layer of 50 µm of lead oxide and titanium dioxide (two of the most common white pigments in ancient artifacts) on a bulk of 300 µm of ceramic material (SiO_2_). In the analysis of the multilayers, due to the restricted flexibility of Geant4 in handling multilayer materials, we analysed at first the penetration depth of the protons in both layers, including the deposited energy in each material. Using the results, we calculated the consequent X-ray generation produced by the protons. After this, we simulated the transmission of the “as produced” K and L lines inside the layers using the electromagnetic packages of Geant4. The samples were irradiated using protons impinging with different incident angles (Θi).

## Results and Discussion

As first step we tried to reproduce the experimental results that were obtained in the proof-of-principle experiment^11^, *i.e.,* by using laser-accelerated protons that were irradiating a homogenous silver target.

**Figure 2:**
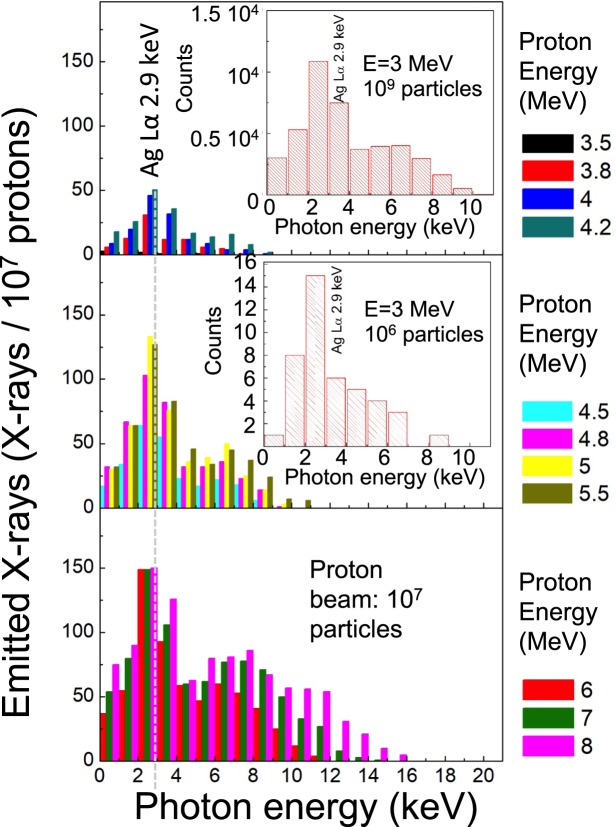
Photon spectra (histograms) for different incident proton energies impinging into a silver surface with incident angle of 80° from the material surface (10° from the target normal) and for a run using 10^7^ particles. The photon energy bin size is 1 keV (as shown in the insets). The figure displays the contribution by the different proton energies within the photon energy bin of 1 keV. In the insets are shown the spectra in the same configuration and for an incident proton with energy 3 MeV and respectively 10^9^ (upper inset) and 10^6^ (lower inset) particles. The vertical dashed grey lines are guides for the eye for identifying the Ag Lα line.

Figure 2 shows the results obtained in the same experimental conditions. For incident proton energies in the range of 4–5 MeV, one can see a ratio of about 46–132 × 10^−7^ photons detected per incident proton for the kα energy of silver, and a value ranging up to 125 × 10^−7^ photons per incident protons for the other photon energies. It should be noted in Fig. 2 that the simulations provide information about the X-ray line Ag Lα which is 2.9 keV, while in the experiments the observed line was the Ag kα (23 keV). This is due to the fact that in the Geant4 simulations we used a Si CCD detector with a good sensitivity for energies lower than 20 keV, while during the experiment we were using a spectrometer that was sensitive only to energies higher than 20 keV. The detector used during the experiment (an ImagePlate of the type BAS2025), had a detection threshold value of 2 × 10^8^ photons/sr^32^. Combining the information about the numbers of particles in the proton beam (around 10^13^ protons/sr/MeV for the energy range 0.5–10 MeV) with the number of silver-produced photons predicted from simulations (10^−4^ photons/proton, using a 1 MeV proton bin around a central energy of 3.5 MeV in the spectrum and integrating the photon yield), we can estimate the number of produced photons in the experiment condition to be in the order of 10^9^ photons/sr, in good agreement with the signal observed in^11^. From Fig. 2 we also see that for proton energies between 5.5–8 MeV, the contribution of photons to the Ag Lα decreases, while these proton energies produce an increased bremsstrahlung. The use of higher proton energies than 5.5 MeV is therefore of very little interest for silver.

It might be noted that the ratio of emitted photons vs impinging protons does not depend on the number of impinging protons: As visible by the two histograms shown as inset of Fig. 2 depicting the photon per proton ratio for simulations using different numbers of protons with mean energy of 3 MeV, the number of detected photons increases proportionally with the particle number. Following this result, we decided to proceed in the subsequent simulations using a proton beam with 10^6^ protons, which can be considered as the best compromise between computational simulation time, yet providing reliable photon information. Given the fact that the particles travel in vacuum and with cylindrical geometry, the distance of the detector and the proton source does not influence these results.

**Figure 3:**
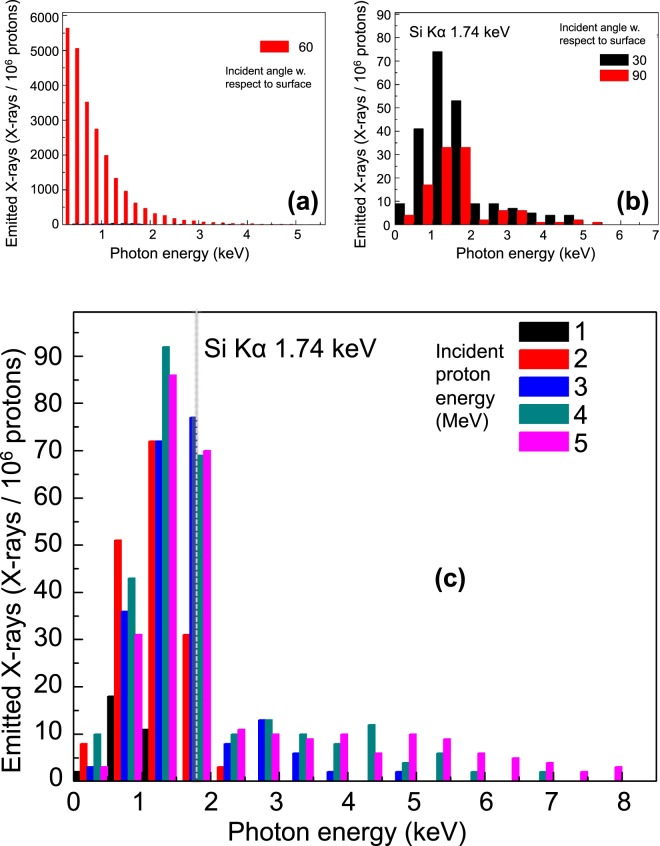
(**a**) Emitted photons (per incident protons) for an incident angle of 60° with respect to the surface for a SiO_2_ material sample; the incident proton energy is 3 MeV and its number 10^6^ protons; (**b**) Emitted photons (per incident protons) for different incident angles (30° and 90°) with respect to the surface for a SiO_2_ material sample, for an incident proton energy of 3 MeV and using 10^6^ protons; (**c**) Emitted photons (per incident protons) for a fixed incident angle of 30° with respect to the surface, for different incident proton energies and 10^6^ protons. Within each photon energy bin (respectively for figure (**a**) 0.25 keV, (**b**,**c**) 0.5 keV), the figure displays the contribution made by the different proton energies. NB related to the panels b and c, considering protons with 3 MeV and 30° incidence: The data refer to two different Monte Carlo simulations, with different random conditions. In particular, the calculations of the bremsstrahlung, the secondary processes and emissions are based on randomly generated information in the algorithms that can produce fluctuations in the results.

As next step, we wanted to identify the best experimental configuration for maximizing the photon production and detection made by the laser-driven PIXE. Firstly, we analysed the impact of the proton incidence angle on the photon emission. To do so, the initial set of simulations was run fixing the proton energy at 3 MeV, one of the most used PIXE proton energies since this energy maximizes some of the materials’ cross-sections. The proton current was set at 10^6^ protons, and the source position at 3 cm from the surface of a homogenous SiO_2_ target. The incidence angle Θi was varied between 30°, 60°, and 90°. The results of these first runs are shown in Fig. 3. Figure 3a, displays the emitted photons per incident proton with mean energy 3 MeV for an incident angle of Θi = 60° with respect to the surface of the X-ray source material (SiO_2_ surface). Simulations show that this angle and below produces the maximum photon detection. However, for this angle, the maximum photon count is obtained for lower photon energies than the material’s kα (located at 1.74 keV), *i.e*., the emitted photons outside this energy range correspond to bremsstrahlung emission. In order to retrieve the best conditions for the generation and detection of the silicon dioxide kα line we performed a zoom on the histograms with lower number of particles and at other angles, *i.e*., for Θi = 90° and Θi = 30°. Figure 3b indicates that the best compromise between reducing the bremsstrahlung emission and enhancing the kα line emission is irradiating the sample with an incident angle around Θi = 30°. Indeed, this lower incidence angle reduces the beam penetration depth, quenching the secondary reactions. These reactions are at the origin of the bremsstrahlung emission. Moreover, the lower volume reached by the proton beam reduces the number of produced photons and consequently the number of secondary recombination effects in the detector. Hence, the angle Θi = 30° was found to be among the best incidence angles for producing a detectable number of kα photons without producing strong bremsstrahlung that can mask the useful signal. When analysing this behaviour for other proton energies of interest in the PIXE, *i.e*., energies ranging from 1–5 MeV (see Fig. 3c), we found the same tendency. This confirmed that – taking into account also different incident proton energies - the best configuration for the incidence angle was around 30°.

Now that we had verified the optimal incident angle, we investigated the photon generation and detection in various homogenous materials at different material depths with steps of about 5 µm and at an optimized incident angle. In this “in-depth analysis”, for a given well-specified proton energy, the detector will detect the X-ray emission from all depths up to the maximum penetration depth. Protons release most of their energy at the end of motion, however, when travelling, they still release some small amount of energy that can produce X-rays. Given the large energy spread of laser-generated protons, laser-PIXE provides access to a broad depth range, but not depth resolution, since it is not possible to differentiate from where the X-rays have been generated. This will require a “layer-by-layer” analysis with more mono-energetic protons (see below).

**Figure 4:**
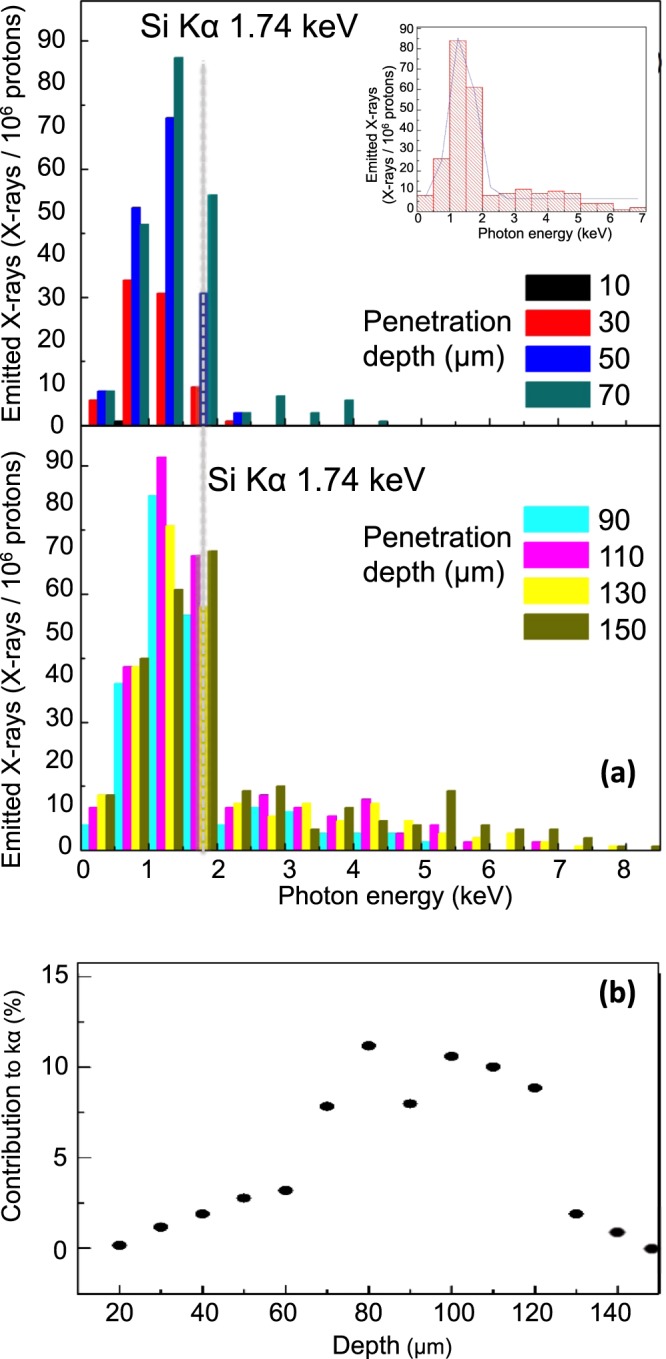
(**a**) Photon spectra for different penetration depths (related to proton with different energies impinging with an incident angle of 30°) for a SiO_2_ sample and for a run using 10^6^ particles (inset: detail of a photon histogramm for a 3 MeV proton incident at 30° on a SiO_2_ material sample); (**b**) total contribution to the kα photon emission as coming from all the penetration layers. For the calculation, only photons within the energy of 1.73–1.75 keV (*i.e.*, very close to the kα) have been considered. This bin size is much smaller than what used in panel (a), where the bin size is 0.5 keV. The kα photon energy position is indicated with a dashed grey line.

Figure 4 illustrates the results related to the analysis of a block of homogenous silicon dioxide material, indicative for ceramic artefact samples. We calculated penetration depth steps of 10 µm, corresponding to an energy step of about 0.3 MeV and using 10^6^ protons. Figure 4a shows the photon histograms, *i.e*., how many photons are emitted from each single material layer where protons deposit their energy, and this for the different incident proton energies. For the sake of clarity the histograms show penetration ranges of about 20 µm (corresponding to about 0.5 MeV energy steps) while the inset shows one single histogram for protons of 3 MeV – corresponding to a depth of 84 µm. From the figure we can see that the number of bremsstrahlung photons increases with the penetration depth of protons: higher energy protons generate more secondary processes, *i.e*., processes that are not specific for the PIXE and that produce bremsstrahlung emission. In addition to this, the histograms show that the number of kα photons (for Si the kα is located at 1.74 keV) increases with increasing penetration depth up to a depth of about 110 µm (equivalent to protons of about 3.0 MeV), and then decreases up to penetration depth of 150 µm (equivalent to proton of about 3.6 MeV). This behaviour can easily be explained calculating the transmission of photons of 1.74 keV (the energy of Si kα) through a silicon dioxide bulk. Following the Bragg principle, which affirms that a proton releases almost all of its energy at a specific energy-dependent depth (end of trajectory), we can approximate the results of a multi-energy irradiation simply by adding the results obtained for different mono-energetic beams. When evaluating the contribution to the total photon spectra of merely the kα photons (energy range 1.73–1.75 keV) produced in each layer (see Fig. 4b), we observe that the protons penetrating in different layers still produce photons that are relevant to the Si kα emission (and detection). Using the electromagnetic test package of Geant4 we can determine the number of transmitted photons that reach the detector. We find a yield of 7–12% for Si kα photons generated at depths of 70–120 µm, below 5% for those produced between 130–150 µm or below 70 µm, and 0.03% for those created at 150 µm (equivalent to proton energies of about 4 MeV). Therefore, photons produced at deeper layers contribute more than those produced by the surface layers. However, these photons are still detectable, also in the classical PIXE. The contribution becomes very low at penetration depths of about 130 µm, when the photons do not have sufficient energy to travel trough the target and as such do not reach the detector. When integration over all penetration depths, the total number of detected photons reaches a value of 10^−3^ photons per incident proton, clearly indicating that the experiments taken with multi-energetic beams enhances the global photon production and detection. From the above shown results, we can assess that laser-driven protons allow a volumetric analysis of a SiO_2_ material up to a penetration depth of 130 µm. This can be achieved using laser-generated protons with energies up to a few (about 4) MeV.

**Figure 5:**
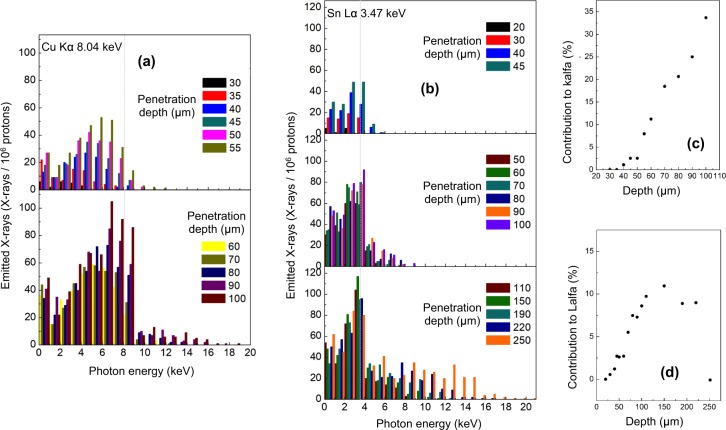
(**a**) Photon spectra for different incident penetration depths (impinging with an incident angle of 30° on a Cu sample); (**b**) Photon spectra for different incident penetration depths (impinging with incident angle of 30°) on a Sn material sample; (**c**) Contribution to the kα detection vs penetration depth within a Cu material sample; (**d**) Contribution to the Lα detection vs penetration depth within a Sn material sample. The Lα/kα photon energy positions are indicated with a dashed grey line in panel (a and b).

Similar results are observed for simulations using a bronze alloy (material which consists of 90% Cu and 10% Sn) shown in Fig. 5a and in Fig. 5b. The histograms obtained for this material show both, the kα line of Cu and the Lα line of Sn. Similarly to the case of silicon dioxide, the bremsstrahlung radiation increases with the proton penetration in the bulk due to an increase in the number of secondary processes.

Figure 5c displays the contribution of the Cu kα line to the total photon production obtained when a proton beam with all energies is impinging the sample. One can see that the contribution constantly increases with increasing proton penetration; this is confirmed by the transmittance simulation, which does not indicate losses in the transmittance of 8 keV photons for a travel distance of 150 µm in bronze. Analysing the contribution of the Sn Lα line to the total photon production when protons with all energies are impinging (see Fig. 5d), one can see that, likewise the kα line of silicon dioxide, the contribution increases until a specific depth (150 µm in this case) and then decreases. The transmittance simulation for the Sn Lα line in bronze indicates that the transmittance strongly decreases after 150 µm (corresponding to a proton energy of 5.8 MeV), reaching very low values of around 0.03%.

Again, one can deduce from the above-obtained results, that the laser-driven protons allow a volumetric analysis of a bronze artefact within a distance of about 150 µm, and the analysis requires protons with energies up to about 6 MeV.

**Figure 6:**
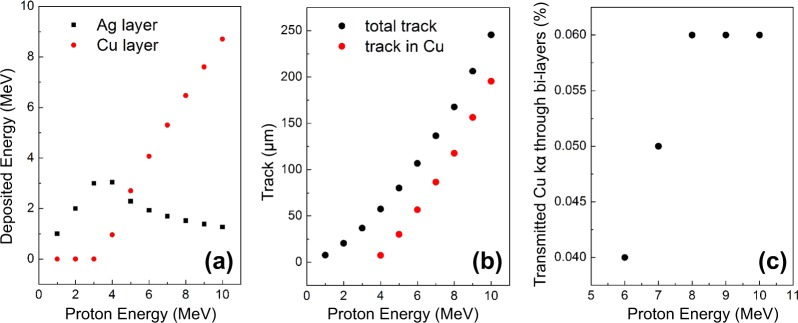
(**a**) Deposited energy vs proton energy for the two materials considered in the multilayer target made of Ag//Cu; (**b**) black dots: penetration depth (“track”) for the impinging protons, with detail of the penetration within Cu; (**c**) transmitted Cu kα through the bi-layers (in %), compared to the total photon production in this layer. Results are obtained for an angle of incidence of 30° and 10^6^ protons.

The last simulations were performed for two multilayer materials. Figure 6 shows the results obtained for an Ag(50 µm)//Copper(300 µm) multilayer sample as function of the proton energy. The data clearly indicate that the proton energy is completely deposited in the Ag layer up to a proton energy of 4 MeV: in this case only the Ag kα line is detected by the detector and the Geant4 simulations produce the same histograms as shown in Fig. 2. For a proton energy higher than 4 MeV the proton beams penetrate into the Cu layer starting the generation of Cu kα photons. However, the number of Cu photons that reach the detector, *i.e*., that are able to cross the bilayer, is lower than those detected for a homogeneous Cu target with the same deposited energy. Simulation of the photon transmittance (Fig. 6b,c) for the Cu kα line only indicate a transmittance ranging from 0.04% (obtained at the Ag//Cu interface) and 0.06% (obtained for a penetration depth in Cu of about 200 – equivalent to an energy of 8 MeV) decreasing the number of detected photons to about 10^−10^ photons per incident proton. For the multilayer sample based on Metal//Silicon dioxide (such as for example Pb//SiO_2_ or Ti//SiO_2_) the transmittance of photons generated in the SiO_2_ layer is extremely moderate (close to zero) due to the very low energy of the SiO_2_ kα photons. A similar behaviour is found for multilayers consisting of Pigments//Ceramic and Pigments//Canvas, simulated as a PbO layer on SiO_2_ (or CaCO_3_ for the canvas), or a TiO_2_ layer on SiO_2_. Again, the transmittance of photons generated in the SiO_2_ layer almost negligible, due to the very low energy of Si and Ca kα photons that are produced in the layer and that are not able to cross the material bulk and reach the detector. As such, even using laser-generated protons, the analysis is difficult in bilayer materials where the kα line is below 6 keV. This is due to the fact that the transmission of the photons within the bilayers is low and would require a photomultiplier to be detected.

## References

[1] Abbott A (2012). Laser centre lights up eastern Europe. Nature.

[2] Antici P (2008). Numerical study of a linear accelerator using laser generated proton beams as a source. J. of Applied Physics.

[3] Yogo A (2011). Measurement of relative biological effectiveness of protons in human cancer cells using a laser-driven quasimonoenergetic proton beamline. Appl. Phys. Lett..

[4] Antici P (2011). A compact post-acceleration scheme for laser generated protons. Phys. Plasmas.

[5] Antici P (2012). Laser-driven electron beamlines generated by coupling laser-plasma sources with conventional transport systems. J. Appl. Phys..

[6] Cirrone P (2015). Design and Status of the ELIMED Beam Line for Laser-Driven Ion Beams. Appl. Sci..

[7] Scisciò M (2016). Parametric study of transport beam lines for electron beams accelerated by laser-plasma interaction. J. Appl. Phys..

[8] Schillaci F (2016). Characterization of the ELIMED Permanent Magnets Quadrupole system prototype with laser-driven proton beams. Journal of Instrumentation.

[9] Scisciò M, Migliorati M, Palumbo L, Antici P (2018). Design and optimization of a compact laser-driven proton beamline. Sci. Rep..

[10] Dromey B (2016). Picosecond metrology of laser-driven proton bursts. Nature Comm..

[11] Barberio, M., Veltri, S., Scisciò, M. & Antici, P. Laser-Accelerated Proton Beams as Diagnostics for Cultural Heritage, *Sci. Rep*. **7**, 40415 10.1038/srep40415 PMC5339728 28266496

[12] Barberio M (2017). Laser-Generated Proton Beams for High-Precision Ultra-Fast Crystal Synthesis. Sci. Rep..

[13] Barberio M (2018). Laser-Accelerated Particle Beams for Stress Testing of Materials. Nat. Comm..

[14] Barberio, M., Scisciò, M., Skantzakis, E. & Antici, P. Carbon based nanostructured film materials for high-intense laser-matter interaction experiments, *Adv. Eng. Mater*. 1800777 (2018).

[15] Barberio M (2018). Graphitization of diamond by laser-accelerated proton beams. Carbon.

[16] http://europa.eu/cultural-heritage/european-year-cultural-heritage_en.

[17] Chiari G (2008). Saving art. in situ, Nature.

[18] Bawaya M (2015). Salvaging Science. Science.

[19] Baglioni P, Carretti E, Chelazzi D (2015). Nanomaterials in art conservation. Nature Nanotechnology.

[20] Vanmeert, F. *et al*. Back Cover: Chemical Mapping by Macroscopic X-ray Powder Diffraction (MA-XRPD) of Van Gogh’s Sunflowers: Identification of Areas with Higher Degradation Risk, *Angew. Chem. Int*., **57**, 1 (2018) - press coverage: https://www.nytimes.com/2018/06/01/arts/design/van-gogh-yellow-sunflowers.html (accessed July 2018). 10.1002/anie.201713293 29498460

[21] https://www.theguardian.com/artanddesign/2017/nov/15/leonardo-da-vinci-salvator-mundi-auction (accessed July 2018).

[22] Malmqvist KG (1986). Comparison between PIXE and XRF for applications in art and archaeology. Nuclear Instruments and Methods in Physics Research Section B: Beam Interactions with Materials and Atoms.

[23] Lekki J (2017). Comparison of PIXE and XRF in the analysis of silver denarii of the early Piast. J. Radioanal. Nucl. Chem..

[24] Lebon M., Pichon L., Beck L. (2018). Enhanced identification of trace element fingerprint of prehistoric pigments by PIXE mapping. Nuclear Instruments and Methods in Physics Research Section B: Beam Interactions with Materials and Atoms.

[25] Agostinelli S (2003). Geant4—A Simulation Toolkit. Nuclear Instruments and Methods in Physics Research A.

[26] Pia MG (2009). PIXE simulations with Geant. IEEE Trans. Nucl. Sci..

[27] Lechner A, Pia MG, Sudhakar M (2009). Validation of Geant4 low energy electromagnetic processes against precision measurements of electron energy deposit. IEEE Trans. Nucl. Sci..

[28] Amako K (2005). Comparison of Geant4 electromagnetic physics models against the NIST reference data. IEEE Trans. Nucl. Sci..

[29] Guatelli S, Mantero A, Mascialino B, Nieminen P, Pia MG (2007). Geant4 Atomic Relaxation. IEEE Trans. Nucl. Sci..

[30] Pia MG, Saracco P, Sudhakar M (2009). Validation of radiative transition probability calculations. IEEE Trans. Nucl. Sci..

[31] Ziegler JF (2004). SRIM 2003. Nuclear Instruments and Methods in Physics Research B.

[32] Schlenvoigt, H. P. Information on the LCS 2 hard X-ray spectrometer Calibration and usage, *LULI internal report* dated November 29, page 22; and private communication (2010).

